# Cultural Differences in Professional Help Seeking: A Comparison of Japan and the U.S.

**DOI:** 10.3389/fpsyg.2012.00615

**Published:** 2013-01-11

**Authors:** Taraneh Mojaverian, Takeshi Hashimoto, Heejung S. Kim

**Affiliations:** ^1^Department of Psychological and Brain Sciences, University of California Santa BarbaraSanta Barbara, CA, USA; ^2^Department of Social and Human Studies, Shizuoka UniversityShizuoka, Japan

**Keywords:** culture, help seeking, professional help, clinical services, social support

## Abstract

Previous research has found cultural differences in the frequency of support seeking. Asians and Asian Americans report seeking support from their close others to deal with their stress less often compared to European Americans. Similarly, other research on professional help seeking has shown that Asians and Asian Americans are less likely than European Americans to seek professional psychological help. Previous studies link this difference to multitude of factors, such as cultural stigma and reliance on informal social networks. The present research examined another explanation for cultural differences in professional help seeking. We predicted that the observed cultural difference in professional help seeking is an extension of culture-specific interpersonal relationship patterns. In the present research, undergraduate students in Japan and the United States completed the Inventory of Attitudes toward Seeking Mental Health Services, which measures professional help seeking propensity, psychological openness to acknowledging psychological problems, and indifference to the stigma of seeking professional help. The results showed that Japanese reported greater reluctance to seek professional help compared to Americans. Moreover, the relationship between culture and professional help seeking attitudes was partially mediated by use of social support seeking among close others. The implications of cultural differences in professional help seeking and the relationship between support seeking and professional help seeking are discussed.

## Introduction

Many problems that people experience in contemporary society, such as bullying in school and the workplace, poverty, domestic violence, and abuse, are difficult to cope with by oneself, and help from others may be a necessity. Professional help providers, including physicians, therapists, and counselors, are potential sources of support, in addition to close others such as friends and family within one’s personal network. In order to receive assistance from these professionals, the person in need must actively solicit their help. If people do not seek help, professional helping systems are ineffective regardless of their availability.

Much research on professional help seeking suggests that there is a systematic difference among people from different cultural contexts in frequency of help seeking. For example, people from Asian and Asian American cultural contexts are less willing to seek out professional help than those from European American contexts (for reviews, see Mizuno and Ishikuma, 1999; Hwang, 2006). With this in mind, understanding the reasons underlying why individuals decide to seek or not seek professional help becomes an important issue to consider, with the goal of both investigating the effectiveness of such help and of maximizing the benefit that people from all cultural experiences can draw from these social structural resources, when and if these resources may be beneficial. In the present research, we examine how cultural differences in relational patterns in everyday social interactions, such as social support seeking, are associated with attitudes toward seeking professional help.

Cultural norms may be a determining factor in attitudes toward seeking professional help. It is reasonable to assume that culture influences several aspects of professional help seeking, including recognition and attribution of problems, decision making for help seeking, and evaluation of various coping resources (Cauce et al., 2002). In particular, differences in relational patterns across cultures have implications for seeking help from professionals. Collectivistic cultures, such as East Asia, emphasize interdependence, and social harmony within the group, with each individual viewed as fundamentally interconnected in a larger social unit (Markus and Kitayama, 1991). By contrast, individualistic cultures, such as the United States, emphasize independence, distinguishing the individual as autonomous and distinct from others, with personal motives superseding group interests (Kwan et al., 1997). These cultural differences in the relative focus of individual autonomy versus connection to others have consequences for appraisal of different coping strategies and resources, as we will discuss in the following sections.

Cultural values and attitudes might influence help seeking propensity (HSP) in two ways, as is the case with all socio-cultural learning. One is enculturation, or the process of being socialized into and retaining cultural norms of one’s heritage culture, and another is acculturation, or the process of adaptation to the norms of majority culture while downplaying the process of retention of one’s heritage cultural norms. For instance, among Asian Americans, a passive attitude toward help seeking might be caused by either socializing to their heritage cultural values that inhibit help seeking, related to enculturation, or non-identification with the mainstream American cultural values which promote help seeking, related to acculturation (Kim, 2007). However, it is not clear what aspects of their heritage cultural shaping could inhibit help seeking. Examining this issue in detail will further understanding and improvement of negative attitudes toward help seeking among people from Asian and Asian American cultural contexts.

The cultural shaping of attitudes toward professional help seeking involves several aspects. In previous research, concern for the stigma surrounding professional help seeking has been examined as an important factor in avoidance of professional help use (e.g., Corrigan, 2004). Research has found families from East Asian heritage cultural backgrounds prefer to deal with issues related to mental illness themselves instead of looking toward mental health professionals for assistance, out of concern for stigma related to mental illness (Lin et al., 1982; Root, 1985). Research has consistently shown that family plays a large role in care and treatment of family members among Asian Americans (Lin et al., 1991; Lin and Cheung, 1999; Park and Chelsa, 2010). However, professional help seeking on the individual level may not be related to support from family sources. In fact, other research has found that family support was not predictive of help seeking for emotional distress among Chinese Americans, such that low support from the family was not related to more frequent professional help seeking (Abe-Kim et al., 2002). Taken together, while the family is a potential source of support, low levels of professional help seeking commonly found among people who engage in Asian and Asian American cultural contexts may not necessarily be due to higher levels of family support.

Although the cultural stigma factor has been the focus of much previous research on professional help seeking, other factors are also of note. Another facet of attitudes toward professional help seeking may be psychological openness (PO), or internalized pressure for self-responsibility and for not acknowledging psychological problems openly (Root, 1985; Masuda et al., 2009). In many Asian cultural contexts seeking help from an out-group source (such as mental health professionals) might itself become a problem by causing discord within the in-group. Given relatively strong values placed on familial obligations in many Asian cultural contexts, the illness of a family member is seen as a potential disruption of family balance, leading to the entire family’s involvement in the care of the individual, where seeking help becomes a family decision rather than a personal decision (Lin and Cheung, 1999). One line of research evaluated the level of family involvement with the treatment of schizophrenic patients, and found that family members of Asian American patients were more intimately involved in the treatment process than Caucasians (i.e., those who are from mainstream American cultural contexts; Lin et al., 1991). These findings suggest that, in Asian cultural contexts, the act of seeking help from professionals might be viewed as involving both the individual and the in-group. Finally, in collectivistic cultures (i.e., Asian Americans), disclosing one’s problems to professionals (as out-group agent) could be interpreted as a result of dysfunction of one’s in-group. This could be seen as a threat to in-group relational functioning. Therefore, people from collectivistic cultures might to be more reluctant to seek professional help than people from individualistic cultures (Root, 1985).

In addition to other important factors in propensity for help seeking, such as stigma concerns, we propose another potential explanatory influence that may also independently contribute to variation in HSP. This factor, which we explore in more detail in the current research, is attitudes toward everyday interpersonal support seeking, as part of more general relational norms about seeking help. While a great deal of research has looked at cultural influences in attitudes toward professional help seeking and related concerns, it may also be helpful to consider professional help seeking as an extension of help sought by close others, such as family and friends. When confronting a problem, people can seek and get help from not only professionals but also their social networks. Seeking help from one’s social networks, or use of social support, has been a topic of cross-cultural research. Previous studies suggest that Asians and Asian Americans are more reluctant than European Americans to seek explicit social support as a coping strategy for dealing with stress (Taylor et al., 2004; Kim et al., 2006) out of concern for negative relational consequences of seeking support such as disrupting group harmony or losing face, even though using support can be more effective for Asians than for European Americans in some cases (Kitayama and Markus, 2000; Morling et al., 2003; Uchida et al., 2008). Additionally, when primed to think of the groups they are close to, Asian Americans rated support seeking as less helpful, while European Americans were unaffected by the prime, suggesting that the perceived utility of support seeking may be related to relational norms and attitudes toward seeking support (Kim et al., 2006). This research suggests that, compared with European Americans, those who are from Asian cultural contexts are more reluctant to seek help and support in general, and this tendency may be applicable to attitudes toward professional help seeking.

Of course, professional help differs in several ways from social support from families and friends. Although arranging a visit to a professional provider may be more difficult (Cortina, 2004; Rickwood et al., 2005), professionals offer advanced skills for problem solving as well as individual confidentiality. Nevertheless, even relationships with clinical professionals are social relationships, and thus, how and why people choose to seek or not to seek professional help could be governed under similar reasons as how and why people choose to seek or not to seek social support from their close others. For example, seeking help from a professional and seeking help from close others may both be considered to be potentially disruptive to the group in Asian cultural contexts, as previous research has found these concerns are a factor in both support seeking and professional help seeking. Thus, attitudes toward social support seeking may be related to attitudes toward seeking professional help, and both attitudes may be shaped by cultural influences on relational patterns about seeking help from others. Previous research has shown less use of both social support seeking and professional help seeking among Asians compared to North Americans. These lines of research have remained largely separate, however, although they may stem from the same cultural values.

The purpose of the present research is to examine a potential process, social support seeking from friends and family, which may explain cultural differences in attitudes toward help seeking from professional help providers. In the current research, comparing participants from Japanese and the U.S. cultural contexts, we examine cultural differences in attitudes related to professional help seeking. We hypothesize that Japanese participants will report less willingness to use professional help seeking, compared to U.S. participants, and that attitudes toward support seeking can explain (mediate) cultural differences in willingness for professional help seeking. We also look at the role of indifference to stigma (IS) of seeking professional help in the relationship between support seeking and professional help seeking attitudes.

## Materials and Methods

### Participants

A total of 289 Japanese undergraduates at two universities in Shizuoka Prefecture (120 males, 169 females, aged 18–26, *M* = 19.74, SD = 0.95) and 144 European American undergraduates at the University of California, Santa Barbara (56 males, 88 females, aged 18–26, *M* = 19.14, SD = 1.33)^1^ completed the questionnaire in exchange for course credit. The Japanese students were all native Japanese. The European American students self-identified as such. All three schools provide free counseling services to enrolled students. Participants in both countries were located in medium-sized cities at a similar distance to larger cities (Tokyo and Los Angeles, respectively).

### Materials and procedure

Participants in both cultures filled out a paper questionnaire that included the Brief COPE and the Inventory of Attitudes toward Seeking Mental Health Services (IASMHS), as well as several scales unrelated to the current research topic. Materials were translated from English to Japanese by a bilingual Japanese and English speaker, and checked for accuracy by two additional bilingual Japanese-English speakers.

Participants first filled out the Brief COPE, where they described what they usually do to cope when they experience stressful events. The Brief COPE (Carver, 1997), a shorter version of the original COPE (Carver et al., 1989), is a measure including 14 coping subscales with two items in each subscale. Participants responded using a five-point scale with 1 indicating *not at all* and 5 indicating *very much*. In this study, we focus on two subscales, emotional support, e.g., “I get comfort and understanding from someone,” and instrumental support, e.g., “I try to get advice or help from other people about what to do,” as a measure of support seeking tendencies from personal relationships. This scale was also used in previous research on culture and social support seeking which revealed cultural differences in support seeking tendencies (Taylor et al., 2004; Kim et al., 2006).

Later in the questionnaire, participants filled out the Inventory of Attitudes toward Seeking Mental Health Services (IASMHS). The IASMHS (MacKenzie et al., 2004), is a measure to assess help seeking tendencies from professionals, i.e., psychologists, psychiatrists, social workers, and family physicians. This scale includes 24 items, and participants indicated their agreement with each statement on a 1 (*disagree*) to 5 (*agree*) scale. The IASMHS scale consists of three subscales. The first subscale is HSP, or the extent to which individuals believe they are willing and able to seek professional psychological help. This subscale includes items such as “If I were experiencing a serious psychological problem at this point in my life, I would be confident that I could find relief in psychotherapy” and “If I believed I were having a mental breakdown, my first inclination would be to get professional attention.” We used this subscale as an index of the tendency for help seeking from professionals. The second subscale is PO, or the extent to which individuals are open to acknowledging psychological problems and to the possibility of seeking professional help for them. This subscale includes items such as “People should work out their own problems; getting professional help should be a last resort” and “People with strong characters can get over psychological problems by themselves and would have little need for professional help” (both reverse-coded), so we used this subscale as a index of preferring self-responsibility. The third subscale is IS, or the extent to which individuals are not concerned about what various important others might think should they find out that the individual was seeking professional help for psychological problems. This subscale includes items such as “Having been mentally ill carries with it a burden of shame” and “I would be uncomfortable seeking professional help for psychological problems because people in my social or business circles might find out about it” (both as reverse-coded items), so we used this subscale as a index of lack of concern of the stigma of seeking professional help.

## Results

### Cultural differences in professional help seeking attitudes

Factor analysis (principal factor method, promax rotation) on the IASMHS indicated validation of the original model for two of the three factors (all items over 0.40 on the assumed model). Reliability of the IASMHS subscales by culture was high in both cultures for HSP (eight items; α = 0.72 in Japan and α = 0.78 in U.S.), and IS (eight items; α = 0.79 in Japan and α = 0.82 in U.S.). Means of the subscale items were used as subscale scores. However, the PO subscale did not have acceptable reliability among Japanese participants (eight items; α = 0.38 in Japan and α = 0.71 in U.S.). Therefore, PO was not used as a factor in subsequent analyses.

Cultural differences in each of the IASMHS subscales were analyzed with a two (gender: male, female) by two (culture: U.S., Japan) analysis of variance. Gender was included in analyses as previous research on professional help seeking has found differences among men and women in their willingness to seek help (e.g., Mackenzie et al., 2006).

#### Help seeking propensity

The gender by culture ANOVA on HSP showed a significant main effect of culture, *F*(1, 429) = 41.99, *p* < 0.001 (U.S. *M* = 3.47, SD = 0.74; Japan *M* = 2.99, SD = 0.67), with U.S. participants reporting greater propensity for help seeking compared to Japanese participants, following our predictions. There was also a main effect of gender, *F*(1, 429) = 10.34, *p* = 0.001 (males *M* = 3.03, SD = 0.72; females *M* = 3.25, SD = 0.67), with females reporting greater propensity for help seeking. The gender by culture interaction was not significant, *F*(1, 429) = 0.50, *p* = 0.48.

#### Indifference to stigma

The analysis of IS revealed a significant main effect of culture, *F*(1, 429) = 6.20, *p* = 0.01 (U.S. *M* = 3.48, SD = 0.85; Japan *M* = 3.27, SD = 0.79), with U.S. participants reporting greater IS compared to Japanese participants, in line with our hypothesis. There was no significant effect of gender, *F*(1, 429) = 0.64, *p* = 0.42, or a significant interaction, *F*(1, 429) = 0.14, *p* = 0.71. These results suggest that the Japanese are more reluctant than Americans to seek support from professionals, reporting less propensity for help seeking and less IS, mirroring results found in previous research.

### Cultural differences in support seeking

Regarding the Brief COPE, there was high reliability among the four COPE social support items (“I try to get emotional support from others” and “I get comfort and understanding from someone” as emotional support items, and “I try to get advice or help from other people about what to do” and “I get help and advice from other people” as instrumental support items). Therefore, we used the sum of the four items as a measure of support seeking (α = 0.89 in Japan and α = 0.91 in U.S). We initially examined emotional and instrumental support separately, but both components yielded similar results. Thus, we report the results using the support composite. On this support seeking score, a gender by culture ANOVA showed a significant main effect of culture, with U.S. participants reporting greater use of social support seeking compared to Japanese participants, *F*(1, 429) = 4.52, *p* = 0.03 (Japan *M* = 13.50, SD = 3.76; U.S. *M* = 14.50, SD = 3.76), mirroring previous research on culture and social support seeking (Kim et al., 2008). There was also a significant main effect of gender, *F*(1, 429) = 56.01, *p* < 0.001 (males *M* = 12.33, SD = 3.81; females *M* = 14.84, SD = 3.43), with females reporting greater social support seeking. Furthermore, the interaction was also significant, *F*(1, 429) = 5.01, *p* = 0.03. Follow-up pairwise comparisons indicated that American females (*M* = 15.91, SD = 3.29) sought support more frequently than Japanese females (*M* = 14.28, SD = 3.38), *p* < 0.001, while the comparable cultural comparison for males was not significant (U.S. *M* = 12.30, SD = 3.41; Japan *M* = 12.34, SD = 4.00), *p* = 0.94. These cultural differences in support seeking are consistent with previous research, and these significant differences showed patterns similar to those for professional support seeking, although an unexpected interaction emerged.

### Mediation analyses

To examine support seeking and IS as simultaneous mediators of culture and professional HSP, a series of bootstrapping analyses with bias-corrected confidence estimates were conducted using the methods described by Preacher and Hayes (2008). In all analyses, culture was coded with Japan = −1 and U.S. = 1.

#### Support seeking and indifference to stigma as simultaneous mediators of cultural differences in professional help seeking propensity

To show the independent effects of social support seeking and IS as mediators of cultural differences in HSP, we ran a mediational analysis with support seeking and IS as simultaneous mediators. First, we tested mediating effects of support seeking on the relationship between culture and professional HSP. The bootstrap results indicated that the total effect of culture on professional HSP [*c* = 0.23, *t*(426) = 6.81, *p* < 0.001] was reduced when support seeking and IS (our proposed mediators) were included in the model [*c*′ = 0.19, *t*(426) = 5.79, *p* < 0.001]. The effects of culture on support seeking [*a* = 0.50, *t*(426) = 2.62, *p* = 0.009] and on IS were both significant [*a* = 0.10, *t*(426) = 2.51, *p* = 0.01], and support seeking [*b* = 0.05, *t*(426) = 5.91, *p* < 0.001] and IS both predicted professional HSP [*b* = 0.21, *t*(426) = 5.52, *p* < 0.001].

**Figure 1:**
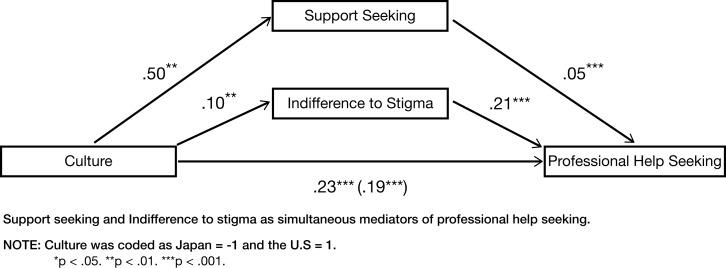
**Mediational analysis**.

Furthermore, the analyses revealed, with 95% confidence, that the indirect effects were significant for both mediators, with a point estimate of 0.02 and a 95% BC (bias-corrected) bootstrap confidence interval of 0.007–0.05 for support seeking, and a point estimate of 0.02 and a 95% BC (bias-corrected) bootstrap confidence interval of 0.005–0.05 for IS. This indicates that both support seeking and IS partially mediated the effect of culture on professional HSP. See Figure 1 for a graphical representation of this mediational analysis.

#### Help seeking propensity as a mediator of cultural differences in support seeking

Given the correlational nature of the current study, it is also important to look at potential alternative models for the relationship between culture, support seeking, and professional help seeking. In order to test a potential alternative model, we reversed the role of support seeking and HSP, testing HSP as a mediator of cultural differences in social support seeking. The bootstrap results indicated that the total effect of culture on support seeking [*c* = 0.57, *t*(426) = 3.26, *p* = 0.001] was reduced when HSP (our proposed mediator) was included in the model [*c*′ = 0.26, *t*(426) = 1.47, *p* = 0.14]. The effect of culture on HSP was significant [*a* = 0.22, *t*(426) = 7.08, *p* < 0.001], and HSP predicted support seeking [*b* = 1.39, *t*(426) = 5.62, *p* < 0.001].

Furthermore, the analyses revealed, with 95% confidence, that the indirect effect was significant, with a point estimate of 0.31 and a 95% BC (bias-corrected) bootstrap confidence interval of 0.21–0.58. This indicates that professional HSP mediated the effect of culture on support seeking.

Reversing the role of support seeking and HSP to test an alternative model, HSP was a significant mediator of cultural differences in support seeking. However, social support seeking is an everyday occurring interpersonal behavior while professional help seeking is a relatively infrequent behavior, and thus, we think the current model is more reasonable. However, statistically, the other model remains a possibility. We address this further in our discussion.

#### Cultural differences in the relationship between indifference to stigma, support seeking, and help seeking propensity

As IS has been introduced in previous research as a potentially important factor, and the current research sought to explore support seeking as an additional factor in the culture and professional help seeking relationship, we were also interested in examining whether the strength of the relationship between IS and professional HSP and the relationship between support seeking and professional HSP were similar in both cultures. We examined whether the association between the IASMHS subscales was comparable for each culture with a series of moderated multiple regressions. In Step 1, we entered IS and culture, *R*^2^ = 0.15, *p* < 0.001. Culture (Japanese = 0, U.S. = 1) was significantly positively associated with IS, β = 0.28, *p* < 0.001. Japanese were less likely to experience IS than Americans. IS was positively related to HSP, β = 0.24, *p* < 0.001. In Step 2, we entered the Culture X IS interaction term, and the interaction was significant, Δ *R*^2^ = 0.18, *p* = 0.003. To interpret this interaction, we calculated the simple slopes between IS and HSP for each culture. IS predicted increased HSP to a greater extent among Americans, β = 0.40, *p* < 0.001, compared with Japanese, β = 0.13, *p* = 0.02.

The association between support seeking and professional HSP in each culture using the same type of analysis shows that the relationship does not significantly differ in both cultures (the interaction was Δ *R*^2^ = 0.001, *p* = 0.59).

## Discussion

Consistent with previous research, cultural differences in attitudes toward professional help seeking were replicated. Japanese were more reluctant than Americans to seek help from professionals, as found in previous research (Mizuno and Ishikuma, 1999; Hwang, 2006). These cultural differences were partially explained by use of support seeking, specifically related to cultural differences in HSP. These results suggest that cultural differences in professional help seeking attitudes are due, in part, to more general attitudes toward support seeking. Comparing the moderating role of culture in the relationship between IS and propensity for help seeking in U.S. and Japanese cultures, it appears as though these attitudes may have a different relationship with each other across cultures. Although there was a positive relationship between HSP and IS in both cultures, this relationship was stronger among U.S. participants compared to Japanese participants. The purpose of the present research was not to test different explanations for professional help seeking as competing factors. In fact, we found that both social support seeking and IS both independently mediated the relationship between culture and professional help seeking attitudes. However, this unexpected cultural difference in the relationship strength between IS and propensity for help seeking suggests that there may be some differences in the relative importance of stigma toward seeking help as a factor affecting propensity for help seeking. Further exploration on this topic is of interest for future research.

We also found effects of gender, particularly for HSP and use of social support. In line with some previous research (e.g., Thoits, 1995; Mackenzie et al., 2006), we found that overall, women reported greater willingness to seek professional help and greater utilization of social support compared to men. These results suggest that gender and cultural differences regarding independence/interdependence may not be the same. While both culture and gender have been shown as sources for relational differences, they may have distinct underlying reasons and mechanisms. Previous research suggests that whether through socialization or biological factors, women tend to develop more socially affiliative tendencies. For instance, gender differences in relational patterns may have not only social and structural but also biobehavioral mechanisms (Taylor et al., 2000), whereas cultural differences appear to be primarily due to engagement within specific socio-cultural contexts (Kim et al., 2008). It is important to note that research on social support, and particularly research on culture and social support, does not find consistent and reliable effects of gender (Waldron and Di Mare, 1998; Neff and Karney, 2005; Kim et al., 2008), and thus, any meaningful theorizing regarding the role of gender in specific social support transactions would require further research, which is a worthwhile topic given its obvious theoretical and practical importance. However, this does highlight the issue of potentially different forms of interdependence. With respect to help seeking norms, there may be more than one way that interdependence shapes support seeking. Interdependence in one context may take the form of relying on others more through the use of social support, whereas in another context, interdependence may take the form of concern with maintaining relational harmony and choosing not to seek support. In the current study, we are looking at a specific way of being interdependent, and these results may not be generalizable in other contexts, including gender. This raises the importance of looking at culture in a multidimensional way.

In the present research, we collapsed emotional and instrumental support seeking into an overall measure of support seeking. However, previous research suggests that there may be cultural differences in the utilization of these two types of support. For example, research comparing emotional and instrumental support provision among European Americans and Japanese found that European Americans reported providing more emotion than problem-focused support, whereas Japanese reported an opposite pattern (Chen et al., 2012). While we did not find differences by support type in the relationship between culture, social support seeking and professional help seeking in the current study, the relationship between these types of support has been shown to be different across cultures in previous research.

It is valuable to recognize that seeking professional help is an explicit coping behavior. Previous research by Taylor et al. (2007) distinguishes between implicit and explicit social support. Implicit social support is defined as emotional comfort one can obtain from social networks without disclosing or discussing one’s problems vis-a-vis specific stressful events, whereas explicit social support is specific recruitment and use of social networks in response to specific stressful events, involving the elicitation of advice, instrumental aid, or emotional comfort. Taylor et al. (2007) found that the use of implicit social support is more beneficial than explicit social support seeking for Asians and Asian Americans. By contrast, European Americans benefited more from use of explicit social support than from implicit social support. This research suggests that among Asians and Asian Americans, effective social support may be less related to talking directly about the problem, and more to do with spending time being around others without talking about the stressor (Kim et al., 2008). If implicit support is more culturally relevant to Asian Americans than to European Americans, and explicit support is more culturally relevant to European Americans than to Asian Americans, explicit support seeking, including seeking professional help, may be seen as more acceptable in American cultural contexts. However, in an Asian cultural context, implicit support from family and friends may be seen more positively than seeking explicit support of any type. Thus, it is important to consider the possibility that professional help seeking, at least in the traditional form, may not be consistent with the dominant cultural model of social relationships and interactions in Asian cultural contexts, and it is not necessarily a problem to resolve. What is viewed as effective for fostering and maintaining mental and physical health may be different depending on one’s cultural context. By exploring the relationship between social support seeking and professional help seeking and looking at how culture may play a role in normative coping behaviors, we seek to better understand and depathologize the lack of professional help seeking in Asian cultures.

Furthermore, rather than emphasizing patient disclosure as part of the treatment process, it may be more helpful for practitioners to take the lead in setting the treatment agenda and structuring discussions with Asian and less acculturated Asian American clients (Sue and Zane, 1987; Hwang, 2006). This change in focus may ultimately encourage greater use of professional services. Research in the domain of social support suggests that explicit received help may be beneficial for people from an East Asian background in some cases if the support is given without previous solicitation. Research has found that Asian Americans reported better outcomes from unsolicited support (support given without prompting from the recipient), compared to support received after active seeking (Mojaverian and Kim, 2013). As such, directive approaches to treatment may be more effective in East Asian cultural contexts.

### Limitations

In this research, we found that cultural differences in professional help seeking attitudes are explained, in part, by differences in the use of support seeking. However, there were some inconsistencies and limitations of this study. Support seeking remained only a partial and not a full mediator of the link between culture and HSP. It is important to note that other factors, such as stigma (also explored in the current research), are still expected to be involved in these cultural differences, as previous research has shown. Additionally, a test of an alternate meditational model, with HSP as a mediator of cultural differences in support seeking, was also statistically supported, suggesting that these two factors are closely interrelated. Our theoretical focus is on support seeking as a mediator of HSP, because it makes more intuitive sense that the pattern of support seeking, an everyday behavior that occurs commonly and generally, would mediate the cultural differences in the professional help seeking, which is a more specific and relatively infrequent behavior. However, given the present research, the directionality of relationships among the key variables remains uncertain. This question necessitates further studies that may more clearly investigate the causal direction of the model.

This study focused specifically on support seeking tendencies in the relationship between culture, social support, and attitudes toward professional help. Looking in more detail about how specific relational concerns surrounding use of social support and concerns about the efficacy of help use would further illuminate this relationship and how it may vary across cultures. Future research with a more fine-grained comparison that includes these factors would be useful in this regard. Additionally, due to the low reliability of the PO subscale of the IASHMS in the Japanese sample, we were not able to include this subscale in our analyses. It is necessary to reconstruct a more culturally sensitive subscale of PO, which has enough reliability and validity for effective cross-cultural comparison.

The current research focused on a comparison between European Americans and Japanese. In the review of previous research related to professional help seeking, we included previous research on Asian Americans to inform our theory regarding Japanese cultural patterns. Our American sample did not include enough Asian Americans to include as a group in our analyses, so we were not able to look directly at Asian Americans in the current study. However, their position is of theoretical interest, as carriers of both American cultural values and Asian cultural values. Previous research by Kim (2007) examined the relationship of enculturation and acculturation to cultural values and attitudes toward seeking professional psychological help among Asian American college students. Enculturation to Asian values showed an inverse relationship to professional help seeking attitudes, above and beyond that of an association with previous counseling experience. On the other hand, a significant relationship was not observed between values of acculturation and professional help seeking attitudes. This result suggests that a negative attitude toward professional help seeking in Asian Americans is due to traditional Asian values that inhibit help seeking, not to lack of European American cultural values. Additionally, factors such as linguistic barriers, perceived cultural relevancy, and lack of knowledge or limited access to professional help services may play a large role for Asian Americans, in contrast to East Asian counterparts (Leong and Lau, 2001). In future research, looking in more depth at the relationship between HSP and culture among Asian Americans would help to inform how holding a bicultural identity may influence utilization of professional help.

Another notable factor is the potential contextual implications of the existing structure of mental health services in the U.S. and Japan. Comparatively, the U.S. has more mental health professionals than Japan, though Japan has more practitioners relative to other East Asian countries (Shinfuku, 1998; Tseng et al., 2001). Greater exposure and availability of mental health services may lead to more positive evaluations of professional help seeking. For example, past research on attitudes toward professional help seeking in Japan and the US has found that previous experience with seeking professional help was associated with more favorable attitudes toward professional help seeking in both cultural contexts (Masuda et al., 2005).

### Implications and future directions

The current research in a novel investigation bringing together previous research on support seeking from close others and research on help seeking from professional help providers, and explores how cultural norms about seeking help may shape both in tandem. From a clinical perspective, the low rates of mental health services use among Asians and Asian Americans in the U.S. is a longstanding topic of interest (see Abe-Kim et al., 2007 for a discussion). The current research suggests that professional help seeking attitudes are related to support seeking, and that cultural values may inform expectations and attitudes about asking for help. Explicitly sharing problems with others, whether within the family or with a professional help provider, may run counter to Asian cultural norms, and accounting for these norms in structuring successful mental health services is important.

The present research underscores the importance of understanding culture-specific meanings of actions, and how these divergent meanings implicate professional help seeking as an extension of patterns of daily behaviors. For example, recognition of the nature of one’s problem, which is assumed as a prerequisite for helping, also affects the likelihood of help seeking behavior to some extent. Robbins (1981) pointed out that there is a consistent tendency for dispositional interpretations (internal attributions) of problems to be associated with use of psychiatric and psychological services. This suggests the possibility that reluctance to seek help among Asians may be due to cultural differences in attribution of problems. That is, Asians may be more likely to make external attributions, whereas European Americans may make more internal attributions (Morris and Peng, 1994), and Asians may therefore evaluate professional help as less useful than do European Americans. In other words, Asians may view their problems as attributable to situational factors rather than to individual factors, and believe it may be difficult to solve their problems by consulting professionals adopting an individual-centered intervention approach.

Moreover, the present research suggests that compared to European Americans, Asians are more concerned about social implications of help seeking in general as behaviors that can negatively impact one’s relationships and social standing, including in the context of professional help seeking. Awareness and understanding of these cultural differences are crucial for successful clinical services. A mismatch between culturally preferred methods of communication and available health services may lead to less use of these services and less effective treatment outcomes when they are used. A focus on disclosing issues and free expression of emotions and concerns found in professional help service frameworks may be a stressor in itself when considering seeking help for Asians (Shea and Yeh, 2008). Research examining factors associated with treatment utilization has found that therapist and client matches in ethnicity are associated with greater length of treatment and lower treatment dropout rates among Asian Americans and other minority groups in the U.S. (Sue et al., 1991). It may be that having a culturally matched practitioner allows for culturally normative communication, improving treatment outcomes.

However, matched practitioner-patient ethnicity may not be the only way to encourage better treatment outcomes. Fostering greater cultural understanding may increase treatment effectiveness regardless of the ethnic background of the practitioner. Recent research has begun to address the need for more culturally informed guidelines for therapy and mental health services (Hwang, 2006, 2011). Creation of ethnic-specific mental health services, which focus on incorporating cultural values into treatment plans and encouraging cultural understanding between mental health professionals and their clients have shown promise, reducing inequities between Asian American and European American treatment outcomes, and increasing service utilization in areas where these services are available (see Leong and Lau, 2001 for a review). Promoting cultural competence among clinical practitioners may encourage more positive attitudes toward seeking professional help and greater use of these services. Understanding how culture informs relational factors on the individual level has widespread implications for cross-cultural mental health beyond the scope of clinical research, assisting in creating effective health resources that allow for people from all cultural backgrounds to receive help when it is needed.

## Conflict of Interest Statement

The authors declare that the research was conducted in the absence of any commercial or financial relationships that could be construed as a potential conflict of interest.

## Footnotes

^1^The initial sample included additional 53 non-European American participants (14 Asian Americans and 39 participants of other ethnicities). The small sample size of Asian Americans did not allow us to include them as a separate group in our analyses.
